# MicroRNAs in the pathophysiology and treatment of status epilepticus

**DOI:** 10.3389/fnmol.2013.00037

**Published:** 2013-11-12

**Authors:** David C. Henshall

**Affiliations:** Department of Physiology and Medical Physics, Royal College of Surgeons in IrelandDublin, Ireland

**Keywords:** argonuate, dicer, epilepsy, epileptogenesis, hippocampal sclerosis, miRNA, non-coding RNA, RNA induced silencing complex

## Abstract

MicroRNA (miRNA) are an important class of non-coding RNA which function as post-transcriptional regulators of gene expression in cells, repressing and fine-tuning protein output. Prolonged seizures (status epilepticus, SE) can cause damage to brain regions such as the hippocampus and result in cognitive deficits and the pathogenesis of epilepsy. Emerging work in animal models has found that SE produces select changes to miRNAs within the brain. Similar changes in over 20 miRNAs have been found in the hippocampus in two or more studies, suggesting conserved miRNA responses after SE. The miRNA changes that accompany SE are predicted to impact levels of multiple proteins involved in neuronal morphology and function, gliosis, neuroinflammation, and cell death. miRNA expression also displays select changes in the blood after SE, supporting blood genomic profiling as potential molecular biomarkers of seizure-damage or epileptogenesis. Intracerebral delivery of chemically modified antisense oligonucleotides (antagomirs) has been shown to have potent, specific and long-lasting effects on brain levels of miRNAs. Targeting miR-34a, miR-132 and miR-184 has been reported to alter seizure-induced neuronal death, whereas targeting miR-134 was neuroprotective, reduced seizure severity during status epilepticus and reduced the later emergence of recurrent spontaneous seizures. These studies support roles for miRNAs in the pathophysiology of status epilepticus and miRNAs may represent novel therapeutic targets to reduce brain injury and epileptogenesis.

## INTRODUCTION

A prolonged, non-terminating seizure (status epilepticus, SE) is a neurological emergency that has potential to cause irreversible brain damage. Uncovering the molecular mechanisms by which seizures transition into an uninterrupted state and elucidating the downstream consequence of such seizures on the brain are important if we are to understand and improve treatment of this devastating condition. MicroRNA (miRNA) have recently been implicated in the pathophysiology of SE and their expressional responses, targets and mechanisms represent a new focus of research in this field with potential to better understand the condition, identify novel therapeutics, and develop diagnostic biomarkers.

## STATUS EPILEPTICUS

Seizures are the result of abnormal, synchronous discharges of groups of neurons in the brain. Most epileptic seizures are self-terminating, often ending within a minute or less (Chen and Wasterlain, 2006). This is thought to be due to homeostatic mechanisms including inactivation of ion channels, build up of the anticonvulsant adenosine within the extracellular space, the anti-excitatory effect of tissue acidosis, and other changes (Lado and Moshe, 2008; Loscher and Kohling, 2010). However, some seizures do not self-terminate. This can result in the development of SE, which is variously defined by duration, often as 30 min of continuous seizure activity or two or more seizures without complete recovery in between. SE can follow drug withdrawal in patients with epilepsy but also occurs due to a myriad of other factors including CNS infection (Tatum Iv et al., 2001). The molecular mechanisms underlying the transition from seizure to SE are poorly understood, but may involve loss of surface receptors for the inhibitory neurotransmitter γ-amino butyric acid (GABA; Wasterlain et al., 2009).

The threshold of impending SE is defined operationally as over 5 min of continuous seizure activity (Chen and Wasterlain, 2006). Such patients require urgent care. Current treatment is with anticonvulsants such as lorazepam or midazolam (intravenous or intramuscular) or certain anti-epileptic drugs including phenytoin (Rossetti and Lowenstein, 2011; Silbergleit et al., 2012). If SE persists, additional combinations may be necessary including intravenous pentobarbital or the anesthetic propofol (Rossetti and Lowenstein, 2011). There is recent clinical evidence supporting the use of the *N*-methyl-D-aspartate (NMDA) receptor antagonist ketamine (Gaspard et al., 2013; Synowiec et al., 2013).

Status epilepticus has the capability of causing profound brain damage. The central mechanism of seizure-induced neuronal injury is glutamate-mediated excitotoxicity but there is also an important contribution from apoptosis-associated signaling pathways (Meldrum, 1991; Fujikawa, 2006; Engel and Henshall, 2009). Status epilepticus also produces synaptic reorganization, gliosis, inflammation, blood-brain barrier (BBB) damage, and lasting changes to excitability (Coulter, 1999; Vezzani et al., 2011). Despite major progress, there remains a need to further improve our understanding of the pathophysiologic mechanisms of SE and explore novel approaches to treatment that may better interrupt SE (particularly pharmacoresistant SE) and prevent long-term deleterious consequences (e.g., provide neuroprotection and anti-epileptogenesis).

## SE TRIGGERS LARGE-SCALE CHANGES IN EXPRESSION OF PROTEIN-CODING GENES

Status epilepticus results in large-scale changes to expression of genes within affected brain regions such as the hippocampus. The most recent microarray analyses in animal models that featured genome-wide coverage found changes to over 1000 genes after SE (Gorter et al., 2006; Jimenez-Mateos et al., 2008; Lauren et al., 2010). Affected biological processes include metabolism, signaling, transport, immune response, transcriptional regulation, cytoskeleton, glial function, neuronal death, and extracellular matrix organization (Lukasiuk and Pitkanen, 2007; Wang et al., 2010; Pitkanen and Lukasiuk, 2011a). There has been recent progress in identifying transcription factors driving up- and down-regulation of protein-coding genes after SE, including Activating transcription factor 5 (ATF5; Torres-Peraza et al., 2013), CCAAT/enhancer-binding protein homologous protein (CHOP; Engel et al., 2013b), Neuron restrictive silencing factor (NRSF/RE1-silencing transcription factor; McClelland et al., 2011) and Nuclear factor erythroid 2-related factor 2 (Nrf2; Mazzuferi et al., 2013). Uncovering the regulatory mechanisms controlling translation of mRNA transcripts represents a largely unexplored aspect of the molecular pathophysiology of SE.

## MicroRNA

MicroRNA represents a potentially critical post-transcriptional mechanism regulating protein levels after SE. miRNA are an endogenous class of small (~23 nt) non-coding RNA that function to regulate gene expression at a post-transcriptional level by targeting mRNAs and reducing protein production (Bartel, 2004). Biogenesis of miRNAs is a highly conserved process which begins with RNA pol II or III-dependent transcription of a primary transcript (pri-miRNA; Lee et al., 2004; Borchert et al., 2006). miRNAs can be transcribed as single units or as part of poly-cistronic miRNA “clusters”, such as miR-17~92 (He et al., 2005) and miR-379~410 (Seitz et al., 2004). The pri-miRNA is processed in the nucleus to a shorter hairpin by the Drosha microprocessor complex (Lee et al., 2003; Gregory et al., 2004). The resulting pre-miRNA is exported to the cytoplasm for further processing by the RNAase III enzyme Dicer. Cleavage of pre-miRNA by Dicer produces the mature miRNA duplex. One strand is selected and incorporated into the RNA-induced silencing complex (RISC) while the other strand is typically degraded.

## HOW miRNAs WORK

**FIGURE 1:**
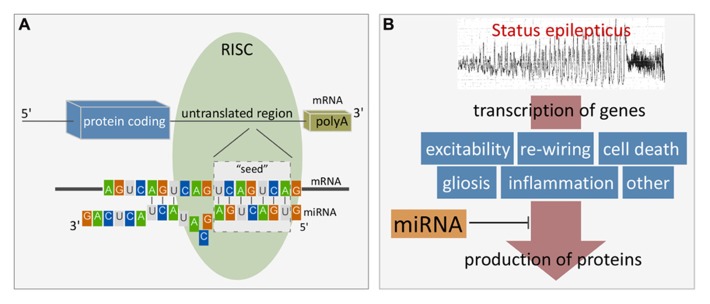
**(A)** Cartoon showing the site and mechanism of miRNA targeting to mRNA. Figure highlights the seven nucleotide “seed” region critical for miRNA binding to the mRNA target. Additional binding also determines specificity and potency. Alignment is facilitated by initial miRNA loading into the RISC which contains Ago2 and GW182 proteins. Binding typically occurs within the 3′UTR. The result is either degradation of the mRNA target or inhibition of translation. **(B)** Cartoon depicting scheme whereby miRNAs influence gene expression after SE. Status epilepticus results in transcriptional up-regulation of 100s of genes. miRNAs lie downstream of this and exert influence over protein production and as a result, influence post-injury outcomes such as repair, cell death and reorganization. Such miRNAs represent potential treatment targets to interrupt pathogenesis of damage and long-term consequences (e.g., hyperexcitability).

MiRNAs control protein output by binding to specific, complementary sequences in target mRNAs of protein-coding genes. MiRNA binding sites are most often found in the 3′untranslated region (UTR) but have also been identified at the 5′end and within the open reading frame (ORF; Bartel, 2009). In mammals, miRNAs usually do not have complete complementarity to the mRNA sequence and therefore do not trigger direct cleavage of the mRNA as occurs with the RNA interference pathway activated by short interfering RNAs (Krol et al., 2010). However, mRNA levels of targets are often reduced by miRNA targeting (Lim et al., 2005; Guo et al., 2010). Targeting involves a 7–8 nucleotide “seed” region within the 5′ end of the miRNA binding to the mRNA via Watson–Crick base-pairing, followed by a variable number of further binding sites (Bartel, 2009; **Figure 1A**). The molecular machinery driving this process is the RISC which is a multi-protein complex, comprising members of the argonaute family as well as GW182 proteins (Fabian et al., 2010). Ago2 is critical in loading the miRNA and bringing it together with the mRNA target. The effect of miRNA targeting of a mRNA can be inhibition of translation or deadenylation and subsequent degradation, or both (Fabian et al., 2010). RISCs containing miRNA and their targets may also be sequestered in processing (P) bodies, including at synapses, which is reversible, enabling later release of the mRNA for translation (Cougot et al., 2008; Saugstad, 2010).

## IMPACT OF miRNAs ON PROTEIN LEVELS

There are over 1500 miRNAs in the human genome (miRBase v19). These are predicted to regulate the levels of at least one third of translated proteins, although over 60% of protein-coding genes are predicted to have miRNA regulatory sites (Friedman et al., 2009). Such extensive control is possible because a single miRNA may be capable of targeting perhaps 200 mRNAs. Not all mRNAs are targets for miRNA, however, and mRNA sequences with short 3′UTRs often lack miRNA binding sites meaning they are probably not significantly regulated in this manner. Conversely, mRNAs with tissue-specific expression or involved in developmental transitions tend to have longer 3′UTRs with more potential miRNA regulatory sites and these transcripts may be under potent miRNA control (Ebert and Sharp, 2012). MiRNAs also display cell and tissue-specific distribution (Lagos-Quintana et al., 2002; Sempere et al., 2004; Shao et al., 2010; He et al., 2012). In the brain, a large number of miRNAs display cell-specific enrichment that contributes to differentiation and distinguishes neurons from astrocytes, oligodendrocytes, and microglia (Jovicic et al., 2013).

The impact of a miRNA on protein levels of its targets is often only within the twofold range (Baek et al., 2008; Selbach et al., 2008). This may fall below the level capable of producing a phenotype, although under conditions of cell stress limited targeting may be enough to produce a larger effect. Multi-targeting of a single mRNA can produce much stronger effects, in the 10-fold range or targeting of multiple mRNAs within the same pathway (Ebert and Sharp, 2012). Nevertheless, for a given miRNA-mRNA pairing, these variables must be determined experimentally and not assumed based on bioinformatics predictions alone. A miRNA strongly predicted to target a particular mRNA may or may not be in a position to influence its translation. This is particularly important when considering the significance of miRNA changes reported in SE studies that analyzed pooled brain regions (e.g., whole hippocampus) containing multiple cell types expressing diverse transcripts and displaying varying degrees of vulnerability to damage after seizures.

## NEURONAL ACTIVITY AND miRNA EXPRESSION

A key function of neuronal miRNAs is in regulating synaptic plasticity in response to neuronal activity (Schratt, 2009; McNeill and Van Vactor, 2012). Dendritic spines are major sites of excitatory communication in the brain and the molecular mechanisms regulating spine size are important determinants of learning, memory, and perhaps, seizure thresholds (Rochefort and Konnerth, 2012). *In situ* hybridization and other visualization techniques reveal enrichment of several miRNAs within dendrites, including miR-134 (Schratt et al., 2006) and miR-138 (Siegel et al., 2009) and neuronal depolarization or glutamate receptor activation results in changes to the expression of these miRNAs. Components of the RISC are also present within the synapto-dendritic compartment (Lugli et al., 2005) and certain dendritically localized miRNAs contain sequences within the precursor form that are identified by chaperone proteins which then shuttle them to the dendrite for later processing to the active mature form (Bicker et al., 2013). The targets of synapto-dendritic miRNAs are involved in dendritic morphogenesis and include p250GAP (by miR-132; Vo et al., 2005; Wayman et al., 2008), Lim kinase (Limk1), and Pumilio2 (by miR-134; Schratt et al., 2006; Fiore et al., 2009) and acyl protein thioesterase 1 (by miR-138; Siegel et al., 2009). This system enables prompt, localized control of neuronal morphology that is responsive to excitatory input (McNeill and Van Vactor, 2012).

## ALTERED microRNA EXPRESSION FOLLOWING SE

MiRNAs may serve important roles in SE by regulating protein production during and after seizures, thereby influencing hyperexcitability, injury, and repair responses (**Figure 1B**). Several studies have profiled miRNA responses in the hippocampus in the acute wake (≤48 h) of SE (Liu et al., 2010; Hu et al., 2011; Jimenez-Mateos et al., 2011; Risbud and Porter, 2013). **Table 1** provides a summary of these studies, including the SE model and profiling platform. Combined, the four studies reveal increased expression levels of approximately 100 different miRNAs after SE while levels of about 200 miRNAs decreased. Although there are difficulties with cross-comparing data derived from different platforms and inter-study differences in the models, seizure severity and timing of sampling, a sub-set of miRNAs changed expression in at least two profiling studies. This includes six up-regulated miRNAs (miRNAs-21, -30c, -125b, -132, -199a, and -375) and nine down-regulated miRNAs (miRNAs-10b, -29a, -98, -181b,c, -374, -381, -450a, and -497; **Table 1**). In another study, miR-10b levels were found to be up-regulated after SE (Jimenez-Mateos et al., 2011). If studies that performed analyses of single miRNAs are also included then this list expands to include up-regulation of miR-34a, miR-134, and miR-155, and down-regulation of miR-9, miR-125a, miR-145, and miR-150 (Hu et al., 2012; Pichardo-Casas et al., 2012; Sano et al., 2012; Ashhab et al., 2013; Peng et al., 2013). Also, miR-146a has been consistently found to be upregulated after SE, albeit at later time points (Aronica et al., 2010; Hu et al., 2012; Omran et al., 2012). Thus, over 20 miRNAs appear to show conserved responses to SE. This is likely an under-estimate, however, since most profiling studies had limited spatio-temporal sampling and incomplete coverage of the rodent miRNAome. The roles of a selection of the profiling-identified miRNAs potentially relevant to seizures and brain function are reviewed briefly below. A potential caveat is that in most cases we do not have direct evidence that these miRNAs are functional – uploaded to the RISC – afterSE.

**Table 1:** miRNA profiling after status epilepticus.

Reference	Platform	SE model	Time point(s) (h)	Profiled	Regulated^a^	Common
Liu et al. (2010)	Taqman	KA (rat)	24	380	31 (13 Up, 18 Down)	Up: miR-21, miR-30c, miR-125b, miR-132, miR-199a, miR-375
Hu et al. (2011)	Microarray	PILO (rat)	24	113	26 (19 Up, 7 Down)	
Jimenez-Mateos et al. (2011)	Taqman	KA (mouse)	24	380	33 (21 Up, 12 Down)	
Risbud and Porter (2013)	Microarray	PILO (rat)	4, 48	All	265 (77 Up, 188 Down)	Down: miR-10b, miR-29a, miR-98, miR-181b,c, miR-374, miR-381, miR-450a, miR-497

*Table summarizes the main studies which have profiled changes to miRNAs in the first 48 h after SE, including the profiling platform, animal model of SE, time point, number of miRNAs studied and the number altered by SE. ^a^Numbers of regulated miRNAs are a guide only – studies differed in terms of what threshold was set for calling a miRNA “regulated” and the application of statistics and/or correction for multiple comparisons. Of note, in the Liu et al study, if miRNAs regulated by at least 1.5-fold are included, then 60 were regulated, with 21 up-regulated and 38 downregulated. Also, miRNAs “not detected” after SE were considered down-regulated in Jimenez-Mateos et al. (2011). Box on far right depicts the commonly regulated miRNAs from these studies (same direction in two or more studies). KA, kainic acid; PILO, pilocarpine.*

### CONSERVED miRNAs DETECTED AFTER SE IN PROFILING STUDIES

Among the four studies to have profiled miRNA responses to SE, miR-132 is the most consistently up-regulated miRNA, being identified in 3/4 of the studies (Hu et al., 2011; Jimenez-Mateos et al., 2011; Risbud and Porter, 2013). Up-regulation of miR-132 was also reported in two other studies that looked at individual miRNA responses to SE (Nudelman et al., 2010; Peng et al., 2013) and miR-132 is over-expressed in human temporal lobe epilepsy (Peng et al., 2013). Increased levels of miR-132 are also present in Ago2-eluted samples from the hippocampus after experimental SE, implying it is functional (Jimenez-Mateos et al., 2011).

The role of miR-132 in the brain is increasingly well understood. Expression of miR-132 is associated with synaptogenesis and a number of miR-132 targets are of potential relevance to the pathophysiology of SE (see **Table 2**). Overexpression of miR-132 in hippocampal neurons in culture was shown to cause neurite (Vo et al., 2005) and dendritic (Wayman et al., 2008) sprouting, and increase excitatory currents (Edbauer et al., 2010). Over-expression of miR-132 *in vivo* (~fivefold was achieved) resulted in an increase in spine density and was associated with a deficit in novel object recognition task (Hansen et al., 2010). In contrast, deletion of miR-132 *in vivo* is associated with decreased dendritic length and branching (Magill et al., 2010) and select defects in synaptic transmission (Pathania et al., 2012). Other predicted targets of miR-132 include MeCP2 (Lusardi et al., 2010), loss of which promotes cognitive deficits, hyperexcitability and seizures (Shahbazian et al., 2002). Targeting of acetylcholinesterase by miR-132 may increase cholinergic tone and influence excitability, hippocampal function, or inflammation (Friedman et al., 2007; Shaked et al., 2009; Shaltiel et al., 2013). The increased hippocampal miR-132 levels that accompany SE and are present in epilepsy may, therefore, influence neuronal morphology and contribute to hyperexcitability or cognitive dysfunction.

**Table 2:** miRNAs targeted in status epilepticus.

miRNA	Targets	Regulatory control	Biological function(s)	Effect of *in vivo* silencing
miR-34a	Bcl-2, CDK4, SIRT1, Map3k9, Syt	p53 (↑), p73 (↑)	Apoptosis, neuronal differentiation	↓ Hippocampal damage, ↓ apoptosis signaling, no change in SE severity
miR-132	MeCP2, P250GAP, AChE	CREB (↑), NRSF (↓)	Dendritic spines (shape, density), ACh breakdown, Gene silencing	↓ Hippocampal damage, no change in SE severity
miR-134	Limk1, Pum2, CREB, DCX	Mef2 (↑), YY1 (↓)	Dendritic spines (shape, complexity), synaptic plasticity, differentiation	↓ Hippocampal damage, ↓ SE severity, ↓ epileptic seizures
miR-184	Akt2, Ago2	STAT3 (↑)	Apoptosis, interleukin signaling	↑ Hippocampal damage, no change in SE severity

*Table lists the four miRNAs which have been targeted *in vivo* in SE models. Table also includes examples of some validated miRNA targets of potential relevance to seizures/epilepsy, examples of their expressional control (transcription factors which act to either increase or decrease levels of the miRNA), examples of established biological functions and, finally, the impact of targeting the miRNA using an LNA-antagomir *in vivo*. AChE, acetylcholinesterase; Ago2, Argonaute 2; Bcl2; B Cell lymphoma 2; CDK4, cyclin-dependent kinase-4; CREB, cAMP-response element binding protein; DCX, doublecortin; Limk1, Lim-domain kinase 1; Map3k9, mitogen-activated protein kinase kinase kinase 9; MeCP2, methyl CpG binding protein 2; p250GAP, Rho GTPase activating protein; Pum2; Pumilio2; SIRT1, Sirtuin 1; Syt, Synaptotagmin; YY1, Yin yang 1.*

Much less is known about the remaining conserved miRNAs from the profiling studies. Most of the functional studies have been in cancer, where their targets have been linked to controlling apoptosis, invasiveness, and cell division. While this may fit with pathways expected to be regulated after SE, it is likely that some (or perhaps most) of the brain targets of these miRNAs in the setting of SE will be different.

Based on available functional studies and known targets, including members of the Bcl-2 family and p53 pathway, increased levels of miR-21, miR-125b, and down-regulation of miR-29a and miR-497 in SE would be expected to have an anti-apoptotic effect (Chan et al., 2005; Mott et al., 2007; Sathyan et al., 2007; Le et al., 2009; Park et al., 2009; Yin et al., 2010; Yadav et al., 2011; Amir et al., 2013). In contrast, down-regulation of miR-10b and miR-98 in SE would be predicted to have a pro-apoptotic effect based on their roles as “oncomirs” (Ma et al., 2007; Ozsait et al., 2010; Foley et al., 2011; Wang et al., 2011). These miRNAs may therefore be involved in the control of apoptosis-associated signaling and regulation of seizure-induced neuronal death (Bozzi et al., 2011; Henshall and Engel, 2013).

Members of the miR-181 family have been linked to promoting cell death (Shi et al., 2008) and may control expression of Bcl-2 family proteins (Ouyang et al., 2012). Astrocytes are particularly enriched in miR-181c, and reduced miR-181b and miR-181c levels promote astrocyte-derived cytokine responses (Hutchison et al., 2013). Thus, miR-181, like miR-146a (Iyer et al., 2012), may negatively regulate inflammatory responses in astrocytes after SE.

For the remaining miRNAs down-regulated in at least two profiling studies – miR-374, miR-381, miR-450a – there is little or no relevant experimental data beyond detection in some cancer models. Whether these represent novel miRNAs with roles in the pathogenesis of SE is uncertain but could be explored in future studies.

## CONTROL OF microRNA EXPRESSION AFTER SE

Although we have an increasingly expansive picture of which miRNAs change after SE and in what direction, we know little about the mechanisms controlling miRNA expression itself. No studies have directly explored miRNA regulatory control in SE, however the transcriptional control mechanism for some SE-regulated miRNAs is understood (**Table 2**). Expression of miR-34a is controlled by p53 (Chang et al., 2007; Raver-Shapira et al., 2007) as well as p73 (Agostini et al., 2011), and an inhibitor of p53 prevented miR-34a upregulation after SE (Sano et al., 2012). Expression of miR-132 is regulated by CREB, a stress-activated transcription factor that promotes neuronal survival (Lee et al., 2009). Some CREB-mediated effects may be pro-epileptogenic. Consistent with this, mice with decreased CREB levels develop fewer spontaneous seizures following pilocarpine-induced SE (Zhu et al., 2012). For miR-134, regulatory control is activity-dependent and driven by Mef2 (Fiore et al., 2009). miRNAs have also been identified under control of the transcriptional repressor NRSF. NRSF is implicated in the epigenetic silencing of multiple genes after SE and interference in NRSF function can recover expression and function of genes whose down-regulation is implicated in epileptogenesis (McClelland et al., 2011). MiR-124 is a known NRSF target that is involved in defining the neuronal phenotype. Levels of miR-124 have been reported to both increase (Peng et al., 2013) and decrease (Risbud and Porter, 2013) following SE in rats. These transcription factors and others may exert their influence on the post-SE molecular environment by modulating expression of miRNAs under their control. Their targeting represents potential approaches for modulating miRNA expression in SE.

Time-course studies have noted abrupt increases in miRNA expression, with rapid turn-on followed by restitution to baseline or lower levels after SE (McKiernan et al., 2012; Sano et al., 2012). This type of precise, dynamic response is reminiscent of miRNA responses during brain development (Krichevsky et al., 2003) and after other CNS insults (Lusardi et al., 2010), and supports tight transcriptional control of the spatiotemporal induction of miRNAs. This has implications for studies where only a single time point has been used because in the absence of a complete time course, erroneous conclusions may be drawn about the full response of a miRNA to SE.

## THERAPEUTIC miRNA TARGETING IN SE

Targeting miRNAs for therapeutic benefit is gaining increasing attention in multiple fields (Brown and Naldini, 2009; Stenvang et al., 2012). If miRNAs exert significant influence over processes involved in either seizure generation or the pathophysiological consequences of SE then miRNA targeting may have therapeutic potential. Delivery of a miRNA inhibitor or replenishment of an otherwise lost miRNA (e.g., via a miRNA mimic) could alter the excitability of the brain leading to less severe seizures or mitigate the downstream consequences leading to neuroprotection.

Brain-expressed miRNAs represent challenging targets for experimental and therapeutic modulation *in vivo*. First, miRNAs display cell-specific expression and tight transcriptional regulation along with their potential for multi-targeting, the control of which is still poorly understood. This means that delivery of an inhibitor or mimic may require careful timing and the means to control where it goes in the brain. Second, small molecules (e.g., <1000 Da) do not yet exist that selectively target miRNAs, although small molecules have been identified which alter miRNA biogenesis (Shan et al., 2008; Melo et al., 2011). A leading approach is to use antisense oligonucleotides (antagomirs; Stenvang et al., 2012). Modifications such as locked nucleic acid (LNA; Wahlestedt et al., 2000) make these potent and selective miRNA inhibitors and further modifications such as placement of cholesterol (Krutzfeldt et al., 2005) or other tags [e.g., penetratin peptide (Schratt et al., 2006)] facilitate cell entry. Studies show that for a miRNA to be inhibited the antagomir must be in several fold excess (Ebert et al., 2007). The mechanism by which antagomirs reduce miRNA function appears to differ depending on the chemistry of the molecules, and includes activation of degradation mechanisms and sequestration as a heteroduplex (Stenvang et al., 2012). Shorter sequences, so-called “tiny LNAs” which share common seed regions of miRNA families may enable blockade of multiple miRNA members and further potentiate targeting efficacy (Obad et al., 2011). A potentially attractive quality of antagomir targeting of miRNAs is prolonged suppression of the miRNA. Silencing of miRNAs by antagomirs has been reported to last several weeks in the periphery (Krutzfeldt et al., 2005; Elmen et al., 2008) and after injection into the brain (Jimenez-Mateos et al., 2012). Another challenge is that antagomirs do not cross an intact BBB (Krutzfeldt et al., 2005). To date, this has been overcome by direct intracerebroventricular microinjection of antagomirs (Jimenez-Mateos et al., 2011; Hu et al., 2012; Jimenez-Mateos et al., 2012; McKiernan et al., 2012; Sano et al., 2012). However, BBB integrity is disrupted by seizures (Marchi et al., 2012) therefore systemic injection may be sufficient to deliver antagomirs into the brain after SE. If the site of BBB disruption is limited to the area of pathologic activity then brain penetration may restrict delivery to the site of injury with minimal effects elsewhere in the brain. Alternatively, strategies can be used to temporarily breach the BBB (Campbell et al., 2008), or antagomirs can be given via intra-nasal delivery (Jimenez-Mateos et al., 2012) or encapsulated in a nanoparticle or exosome (Alvarez-Erviti et al., 2011).

Clinical trials are now underway using antagomirs for non-CNS conditions. Miravirsen targets miR-122, which is involved in hepatitis C virus replication and miravirsen was shown to be safe and effective in patients (Janssen et al., 2013). This raises the possibility of using miRNA-based therapeutics for other diseases, including CNS applications (Brown and Naldini, 2009; Stenvang et al., 2012).

## miRNA TARGETING IN SE

Four miRNAs have been targeted *in vivo* in experimental models of SE using antagomirs (**Table 2**). The first to be targeted was miR-132 (Jimenez-Mateos et al., 2011). Intracerebroventricular injection of an LNA-modified antagomir targeting miR-132 reduced hippocampal levels of miR-132 when measured 24 h later in mice. Animals in which miR-132 had been silenced and then subjected to SE were found to have significantly less damage to the CA3 subfield of the hippocampus (Jimenez-Mateos et al., 2011). No effects of the antagomirs were reported on seizure severity. The mechanism of the protection is unknown and while miR-132 was confirmed in other experiments to be increased in the RISC, the mRNA targets in the RISC were not explored (Jimenez-Mateos et al., 2011). Whether the neuroprotection has any functional effects is unknown as cognitive tests have not yet been performed. However, a similar degree of neuroprotection in the same model was associated with fewer spontaneous seizures in long-term EEG monitoring studies (Jimenez-Mateos et al., 2008; Engel et al., 2010).

Silencing of two other miRNAs has been reported to alter seizure-induced neuronal death without affecting the severity of SE (**Table 2**). MiR-34a is another miRNA whose up-regulation has been reported in multiple models (Hu et al., 2012; Sano et al., 2012). Increased miR-34a levels promote apoptosis via suppressing anti-apoptotic proteins including Bcl-2 (Hermeking, 2010). However, the pro-apoptotic effect of miR-34a in neurons has been questioned (Agostini et al., 2011). More recently, miR-34a was shown to be a positive and negative regulator of neuronal differentiation, targeting synaptotagmin-1 (Agostini et al., 2011), and Numb1 (Fineberg et al., 2012). Inhibition of miR-34a using antagomir infusions into the ventricle of rodents was reported to reduce seizure-induced neuronal death in one study (Hu et al., 2012), but not in another (Sano et al., 2012). In contrast, targeting miR-184, a miRNA up-regulated by a protective episode of brief, non-harmful seizures, resulted in increased susceptibility to seizure-induced neuronal death in mice (McKiernan et al., 2012). Again, seizure severity was not affected. This supports miR-184 having protective effects against seizure-damage, although no candidate targets of this miRNA are obvious to explain this action (**Table 2**). Together with results of miR-132 and miR-34a, these studies reveal miRNAs as potential targets for modulating cell death after SE.

MiR-134 is another activity-regulated miRNA that has been found to be upregulated after SE in kainate and pilocarpine models of SE (Jimenez-Mateos et al., 2011, 2012; Peng et al., 2013). Levels of miR-134 were also confirmed to be increased in the RISC in Ago2 pull down experiments after SE and there were lower protein levels of two validated targets (Jimenez-Mateos et al., 2012). Targeting miR-134 using intracerebroventricular injections of LNA-modified antagomirs produced silencing of the miRNA lasting several weeks. When mice were injected with the antagomirs 24 h before SE, the resulting seizure severity was strongly reduced. Indeed, the seizure suppression was qualitatively similar to the effect of the anticonvulsant lorazepam in the same model (Jimenez-Mateos et al., 2012). Hippocampal damage in these antagomir pre-treated mice was also strongly reduced, although this may have been secondary to the anticonvulsant effect rather than a direct neuroprotective action.

In further experiments, the authors tested the effect of the antagomirs on the development of epilepsy. Antagomirs were injected 1 h after SE, ensuring the initial brain insult was similar between antagomir and scrambled-control SE mice. In EEG and video monitoring of the mice the antagomir-injected animals displayed ~90% fewer spontaneous seizures during the next month (Jimenez-Mateos et al., 2012). Seizure frequency remained reduced 2 months later indicating, presumably, a permanent protective effect. Chronic pathologic changes to the hippocampus including progressive neuronal loss, gliosis, and synaptic reorganization were also reduced (Jimenez-Mateos et al., 2012). The mechanism by which silencing miR-134 produces these strong anti-seizure effects is unknown, although *in vitro* experiments suggested they may be Limk1-dependent (Jimenez-Mateos et al., 2012). These findings suggest antagomirs targeting this miRNA could have neuroprotective and disease-modifying effects which might be a new therapeutic strategy for SE.

## miRNAs AS BIOMARKERS OF SEIZURE-DAMAGE AND EPILEPTOGENESIS

MiRNAs have been recognized as having potential as non-invasive biomarkers (Scholer et al., 2010; de Planell-Saguer and Rodicio, 2011). Unique expression profiles of miRNAs have been reported in blood and other biofluids in animal models and patients and these may be useful as diagnostics, helping to discriminate between diseases with a similar clinical presentation, provide better stratification of patients, predicting disease course, and responses to therapy. Biofluid miRNA profiles could also have applications in toxicology and as markers of tissue damage. Part of their attraction lies with the conserved and widespread function of miRNAs in cell physiology and disease but there are also physico-chemical properties of miRNAs that make them suitable biomarkers. Unlike most other forms of RNA, miRNAs are remarkably stable in biofluids, remaining detectable in serum for weeks and they are also resistant to freeze-thaw and pH changes (Chen et al., 2008; McDonald et al., 2011; Blondal et al., 2013). The stability is attributable, at least in part, to binding to Ago2 (Arroyo et al., 2011; Turchinovich et al., 2011) and presence in membrane-enclosed circulating microvesicles such as exosomes (Hunter et al., 2008; Gallo et al., 2012).

There is an emerging consensus that biomarkers would be useful diagnostics in epilepsy (Pitkanen and Lukasiuk, 2011b; Simonato et al., 2012; Engel et al., 2013a). Molecular biomarkers of SE could be used to gauge insult severity, prognosis, and inform the choice of anticonvulsants or administration of anti-epileptogenic treatments, were they to become available. Evidence is emerging that miRNA signatures in biofluids can distinguish between different forms of neurological disease or acute brain injuries (Rong et al., 2011; Balakathiresan et al., 2012; Baraniskin et al., 2012; Haghikia et al., 2012). To date only a single study has looked at miRNA changes in the blood following SE (Liu et al., 2010). This revealed that kainate-induced seizures in rats produce unique miRNA expression profiles in blood that are different from those produced by other acute neurological injuries, including stroke, and hemorrhage (Liu et al., 2010). The study reported up-regulation of 15 miRNAs and decreased levels of 43 miRNAs in blood, although none passed correction for multiple comparisons (Liu et al., 2010). Nevertheless, this supports biofluid miRNA changes as a source of molecular biomarkers of SE. Notably, a number of the commonly regulated miRNAs identified in the hippocampus in profiling studies are found in serum and plasma, including miR-29a, miR-125b, and miR-375 (Blondal et al., 2013). Just as significantly, several miRNAs increased by SE in the hippocampus are not normally present in these biofluids, including miR-132 and miR-134 (Blondal et al., 2013), supporting their detection post-SE as a potential biomarker of seizures or injury.

## REMAINING CHALLENGES

What are some of the future challenges? There is a need to identify the targets of seizure-regulated miRNAs. This could be achieved using techniques such as HITS-CLIP, whereby RISC-loaded RNAs are cross-linked to proteins followed by Ago2-immunoprecipitation and sequencing (Chi et al., 2009). Knowledge of the *in vivo* targets of miRNAs in SE models would also lead to better understanding of the mechanisms by which antagomirs produce their effects. The specificity of antagomirs in the brain has yet to be established, although some studies have looked at potential off-target effects (Jimenez-Mateos et al., 2011, 2012). Future studies should explore ways to deliver antagomirs via systemic routes while also including assessment of cognitive effects of miRNA silencing. This is particularly relevant for miR-132 and miR-134 because these directly regulate dendritic spines and small changes to levels of miRNA regulating dendritic spines have been found to produce behavioral phenotypes (Hansen et al., 2010).

There are several directions that could be taken to explore the potential of miRNAs as biomarkers in SE. For example, comparing profiles in different biofluids or between different models, and identifying miRNAs with predictive value for epileptogenesis. More clinical data are needed. For example, are miRNAs profiles in the brain or biofluids altered following SE in patients?

Several of the other commonly regulated miRNAs have yet to be targeted in animal models but likely represent focuses of the future. Combinations of miRNA targeting or delivery could offer ways to more completely block deleterious consequences of SE such as epileptogenesis. Experiments could also explore whether antagomirs can have effects on already established epilepsy. Can a disease-modifying effect be produced once epilepsy is established?

Clearly, miRNA functions are directly relevant to seizure thresholds, but clinical applications of miRNA-based therapeutics for SE would most likely be as disease-modifying post-treatments rather than acute anticonvulsants. This is because antagomirs take time to produce miRNA knockdown and measurable effects on the de-repressed targets. This means they are not realistic prospects for stopping SE, although perhaps there would be an application in super-refractory SE (Shorvon, 2011). Nevertheless, faster or more efficient targeting tools may emerge or indeed we may simply identify better miRNA targets or find ways to target the proteins under their control.

## SUMMARY

MiRNAs represent a major additional layer of gene expression control in SE, regulating protein levels within cells in the seizure-damaged brain. As functional studies begin to explore the importance of individual miRNAs in SE we are seeing influences on neuronal death, excitability, gliosis, and neuroinflammation. Many or even most processes dysregulated after SE may be controlled to some degree by miRNA expression. The arrival of miRNA-based inhibitors in clinical trials in other diseases herald translation to the clinic that may eventually also be possible for SE. Translation will be facilitated by focusing on the most critical miRNAs, identifying the molecular targets of miRNAs altered by SE and exploring antagomir delivery routes. Last, miRNAs represent an interesting class of biomarker that may have applications for tracking the severity of injury after SE and whether or not a patient is at risk of long-term consequences such as development or exacerbation of epilepsy.

## Conflict of Interest Statement

The authors declare that the research was conducted in the absence of any commercial or financial relationships that could be construed as a potential conflict of interest.
